# Supplementary material for: The Phylogeography of Deciduous Tree Ulmus macrocarpa (Ulmaceae) in Northern China
Source: Plants (Basel). 2024 May 12;13(10):1334. doi: 10.3390/plants13101334 (PMC11125379; doi:10.3390/plants13101334)
Supplement: Supplementary file 1 [file plants-13-01334-s001.zip › Supplementary Figure.pdf]

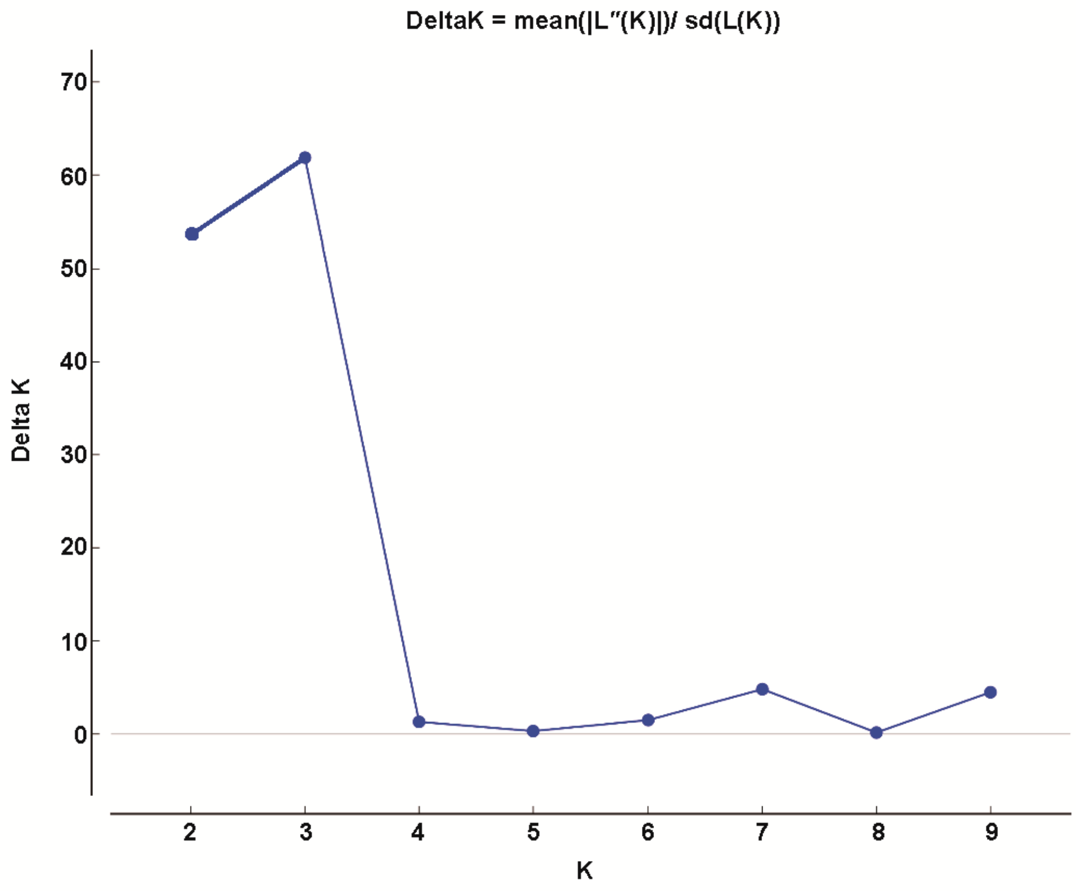

Figure S1. The ΔK plot of structure analysis indicated that the optimal grouping number was K = 3, followed by K = 2.
